# Effect of high-intensity interval training on cardiorespiratory in children and adolescents with overweight or obesity: a meta-analysis of randomized controlled trials

**DOI:** 10.3389/fpubh.2024.1269508

**Published:** 2024-01-26

**Authors:** Yiran Deng, Xianliang Wang

**Affiliations:** School of Physical Education, Shandong University, Jinan City, China

**Keywords:** high-intensity interval training, obesity, children and adolescents, cardiorespiratory fitness, dose–response relationship

## Abstract

**Objective:**

This meta-analysis aimed to examine the effect of high-intensity interval training on cardiorespiratory fitness in children and adolescents with overweight or obesity, and to explore the optimal dose of high-intensity interval training to improve cardiorespiratory fitness in children and adolescents with overweight or obesity.

**Methods:**

Randomized controlled trials on the effects of HIIT on cardiorespiratory fitness in children and adolescents with overweight or obesity were retrieved from six electronic databases, including PubMed, Web of Science, Cochrane Library, CNKI, Wanfang, and VIP. The quality assessment of the included studies was conducted following the revised quality evaluation method based on the PRISMA principles. Keywords for literature search mainly include high-intensity interval, cardiorespiratory fitness, overweight, obese, children, and adolescent, etc.

**Results:**

(1) A total of 18 studies, comprising 581 participants (288 in the intervention group and 293 in the control group), were included and all of them were of moderate to high quality. (2) HIIT had a positive effect on the cardiorespiratory fitness levels of in children and adolescents with overweight or obesity (SMD = 0.91; 95% CI: 0.66, 1.15; *p* < 0.00001). (3) The improvement in cardiorespiratory fitness was more significant when the HIIT intervention lasted for more than 10 weeks (SMD = 1.04; 95% CI: 0.74, 1.34; *p* < 0.00001), was conducted 3 times per week, with 2 to 8 sets per session (SMD = 1.13; 95% CI: 0.71, 1.55; *p* < 0.00001), and maintained a ratio of approximately 1:1 between exercise and rest intervals (SMD = 1.11; 95% CI: 0.73, 1.50; *p* < 0.00001).

**Conclusion and recommendations:**

(1) Long-term HIIT can improve cardiorespiratory fitness in children and adolescents with overweight or obesity. (2) To achieve significant improvements in cardiorespiratory fitness in a short period, children and adolescents with overweight or obesity can engage in HIIT programs lasting for more than 10 weeks, conducted 3 times per week, with 2 to 8 sets per session, and a ratio of approximately 1:1 between exercise and rest intervals.

**Systematic Review Registration:**

Identifier: INPLASY202350033.

## 1 Introduction

Childhood and adolescent obesity and overweight have become a global public health concern. Severe obesity and overweight significantly reduce an individual’s cardiorespiratory fitness (CRF). CRF, as one of the important indicators of physical health, is crucial for maintaining overall health and quality of life. Numerous studies have demonstrated a close association between low CRF levels and increased all-cause mortality rates and the incidence of various cancers (1, 2). Additionally, children and adolescents in their developmental stage who experience prolonged low levels of cardiorespiratory fitness are at a higher risk of cardiovascular and metabolic diseases in adulthood (3, 4). Over the years, Traditional long-duration aerobic exercise (similar to a marathon) has been regarded as a crucial means to reduce fat and promote cardiorespiratory health. Engaging in over 60 min of physical exercise daily can help maintain healthy cardiorespiratory function, and when combined with dietary control or medication, it produces a potent synergistic effect on improving cardiorespiratory fitness and physical health levels in children and adolescents. However, due to high academic pressure and increased entertainment distractions, modern children and adolescents find it challenging to allocate dedicated time for physical exercise beyond school physical education classes. This is where high-intensity interval training (HIIT) comes into play as a time-efficient, highly effective, and rapidly impactful exercise modality, gradually gaining popularity among the general population.

High-intensity interval training (HIIT) is a form of exercise training that involves brief periods of intense, maximal, and explosive movements followed by lower-intensity exercise or rest intervals for recovery (5–7). Previously, HIIT has been proven to have a significant effect on obesity intervention and cardiorespiratory fitness improvement in adults as an emerging, efficient, and time-saving exercise method (8, 9). Existing research suggests that the mechanisms by which HIIT influences body fat content and cardiorespiratory function may involve enhanced fat oxidation after high-intensity exercise and an increase in the secretion of certain hormones. For example, studies by Burgomaster (10) and Talanian (11) have demonstrated that HIIT can increase fatty acid oxidation capacity, thereby accelerating fat consumption to achieve weight control. Additionally, the substantial secretion of catecholamines during HIIT exercise is also a crucial factor in driving the reduction of visceral fat. Another significant mechanism is that HIIT may reduce energy intake by suppressing appetite, consequently decreasing fat accumulation (12). In recent years, related studies and systematic reviews have indicated that HIIT has a beneficial impact on improving cardiorespiratory fitness in obese or overweight children and adolescents (13–15). There is a strong correlation between high levels of childhood obesity, elevated blood pressure, decreased cardiorespiratory fitness, and a lack of physical activity (16). Compared with traditional sports programs, HIIT is short-term, interesting and acceptable. It is a method that can efficiently improve the physical fitness and cardiorespiratory fitness of children and adolescents. Therefore, it is particularly important to explore the effect of high-intensity interval training on cardiorespiratory fitness in children and adolescents with overweight or obesity, and the optimal dose of high-intensity interval training to improve cardiorespiratory fitness in children and adolescents with overweight or obesity.

Currently, although the positive impact of high-intensity interval training on cardiorespiratory health levels has been confirmed (17–19), and there have been controlled experiments and systematic reviews on children and adolescents with overweight or obesity (20–22), the results of the above studies have not reached a consensus. Specifically, there is significant variation in the intervention effects of high-intensity interval training on cardiorespiratory fitness in children and adolescents with overweight or obesity. For example, Abdessalem Koubaa and Brooke Starkoff found that the CRF of participants in the HIIT group was only 10 to 14% higher than that of the control group at the end of their experiments, while Wing Chung Patrick Lau’s research showed that the HIIT group had a CRF value nearly 25% higher than the control group. We believe this discrepancy is closely related to differences in exercise protocols and intervention dosages in experimental designs. Additionally, with the evolution of high-intensity interval exercise, numerous recent studies have emerged on the impact of high-intensity interval training on cardiorespiratory fitness in children and adolescents with overweight or obesity. However, none of these studies have been included in a new systematic review or meta-analysis to expand our understanding of the effects of high-intensity interval training on cardiorespiratory fitness in this population. Furthermore, the existing research has not identified the optimal dose–response relationship of high-intensity interval training in improving cardiorespiratory health levels in children and adolescents with overweight or obesity.

Therefore, this study employs a Meta-analysis method, ensuring methodological quality, to quantitatively analyze randomized controlled trials involving high-intensity interval training for improving cardiorespiratory health levels in children and adolescents with overweight or obesity. The main objectives are to explore two key questions: (1) the overall impact of high-intensity interval training on improving cardiorespiratory fitness in children and adolescents with overweight or obesity, and (2) the optimal dose–response relationship of high-intensity interval training in enhancing cardiorespiratory health levels in children and adolescents with overweight or obesity. This study aims to provide objective evidence for future physical practice and health interventions aimed at improving cardiorespiratory health levels in children and adolescents with overweight or obesity.

## 2 Research methods

### 2.1 Literature search

Six electronic databases, including PubMed, Web of Science, Cochrane Library, CNKI, Wanfang, and VIP, were selected to search for randomized controlled trials (RCTs) on the effects of high-intensity interval training (HIIT) on cardiorespiratory fitness in overweight and obese children and adolescents. The search period ranged from the inception of the databases to March 31, 2023. A combination of subject terms and free-text terms was used to construct the search strategy. The English search strategy was as follows: (“high-intensity interval” OR “high-intensity interval” OR “high intensity intermittent” OR “high-intensity intermittent” OR HIIT) AND (“cardiorespiratory fitness” OR “maximal oxygen uptake” OR “peak oxygen uptake” OR CRF) AND (“children” OR “adolescent”) AND (“obese” OR “overweight”). The Chinese search strategy was as follows: SU = (“High-intensity interval training “+ “HIIT”) AND SU = “Children” + “adolescents” + “pediatric” AND SU = (“obesity” + “overweight”) AND SU = (“cardiorespiratory fitness” + CRF). A total of 1,676 relevant articles were retrieved from the electronic databases. After removing 647 duplicate articles, 1,049 articles remained for further analysis.

### 2.2 Inclusion and exclusion criteria

Two researchers independently assessed the inclusion and exclusion of articles. In cases of disagreement, a third researcher was consulted to reach a consensus through group discussion. The following criteria were used to include studies:

Population: the participants were children and adolescents with overweight or obesity (aged 7–17 years) without prior professional training; The participants did not have acute or chronic diseases.

Intervention: the intervention in the experimental group was HIIT.

Comparison: the intervention in the control group was normal routine or traditional aerobic training.

Outcome: the primary outcome was VO2max. The outcome measures included VO2max or tests from which VO2max values could be indirectly calculated; Secondary outcomes are duration of intervention, intervention period, frequency of intervention, etc.

Study design: published randomized controlled trials (RCTs) were eligible for inclusion, and nonrandomized studies were excluded.

### 2.3 Literature screening

**Figure 1:**
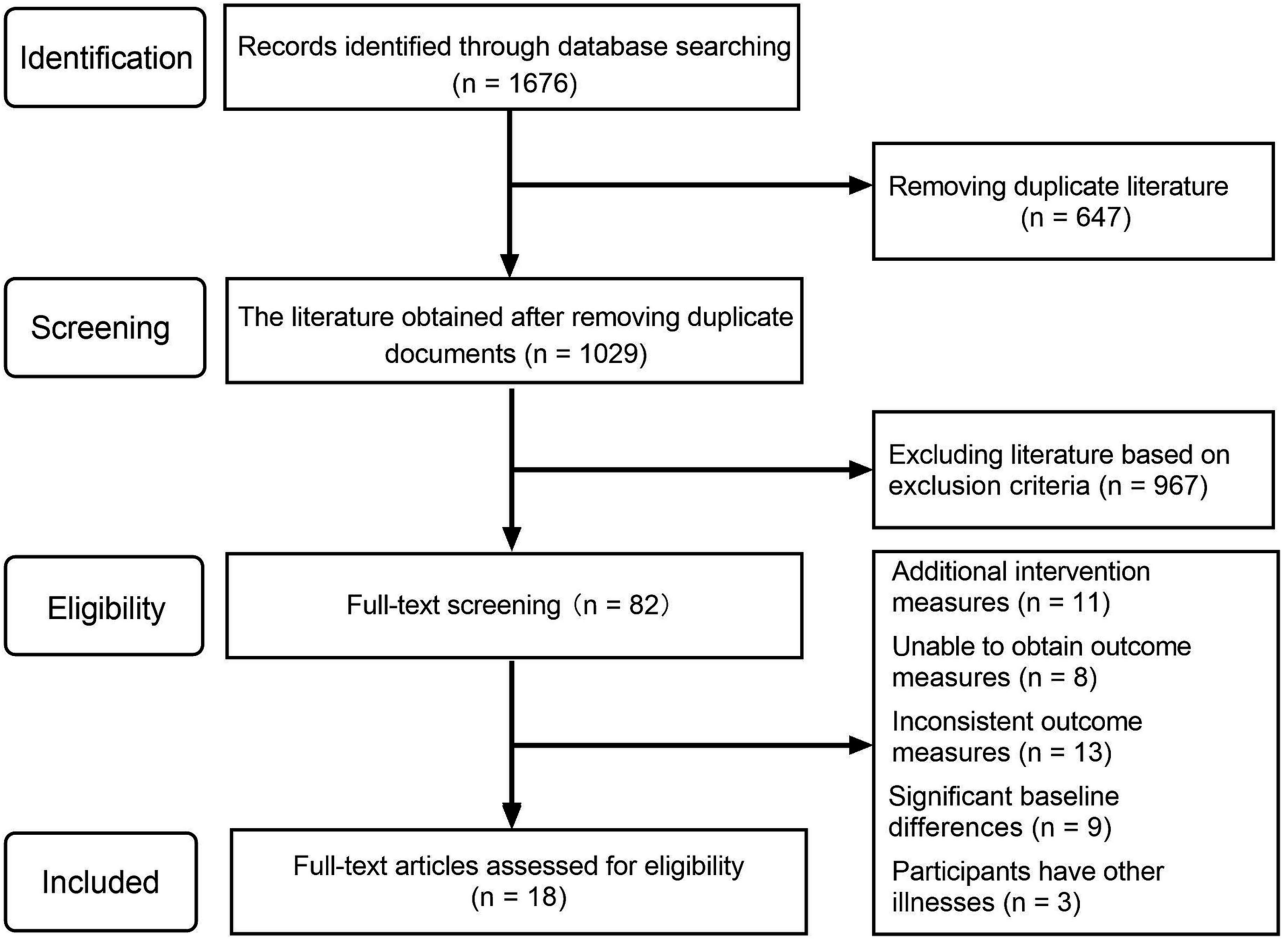
PRISMA flow diagram.

Based on the search and removal of duplicates, 1,049 articles were obtained. Two researchers screened the titles and abstracts and conducted a full-text review, following the inclusion and exclusion criteria. A total of 1,011 articles were excluded, and ultimately, 18 articles were included in the meta-analysis. The process of literature screening is shown in Figure 1.

### 2.4 Characteristics of included studies

The included 18 studies were coded for their characteristics using Excel software. The following features were extracted: author, publication year, sample size, participant age, intervention dose (load time, rest time, number of intervention sets, intervention frequency, intervention duration), and outcome measures (as shown in Table 1). All 18 included studies were RCTs published after 2013. The total sample size of the studies was 581, with 288 participants in the intervention group (ranging from 7 to 23 participants per group) and 293 participants in the control group (ranging from 6 to 26 participants per group). The number of intervention sets varied from 2 to 20, and the intervention duration ranged from 4 to 24 weeks. The intervention frequency was consistent across all studies, with interventions conducted 3 times per week. Regarding outcome measures, except for three studies (Lau, Bogataj, Cvetkovic) that used the YO-YO test to indirectly estimate VO2 max, the remaining 15 studies directly measured VO2 max. The unit of measurement for VO2 max in all included studies was ml/min/kg.

**Table 1:** Basic characteristics of included literature.

Author	Sample size(E/C)	Age (M ± SD)	Intervention dosage				outcome measure
			Load time (intensity)	Rest time (intensity)	number of sets	wk	
Dias (23)	48 (22/26)	E:12.4 ± 1.9\u00B0C:11.8 ± 2.4	4 min (85-95%HRmax)	3 min (50%70%HRmax)	4	12	①
Ingul (24)	48 (22/26)	7–16	4 min (85-95%HRmax)	3 min (50-70%HRmax)	4	12	①
Kargarfard (25)	20 (10/10)	12.36 ± 1.34	4 min (80-90%HRmax)	2 min (40-50%HRmax)	NR	8	①
Murphy (26)	13 (7/6)	E:13.7 ± 2.0\u00B0C:14.3 ± 1.2	1 min (80-90%HRmax)	2 min (60%HRmax)	10	4	①
Starkoff (20)	27 (14/13)	E:14.9 ± 1.6\u00B0C:14.5 ± 1.4	2 min (90-95%HRmax)	1 min (55%HRmax)	10	6	①
Lau (22)	27 (15/12)	E:11.0 ± 0.6\u00B0C:9.9 ± 0.9	15 s (120%MAS)	15 s (Rest)	12	6	②
Racil (27)	23 (11/12)	E:15.6 ± 0.7\u00B0C:15.9 ± 1.2	30s (100-110%MAS)	30s (Rest)	12–16	12	①
Racil (28)	42 (23/19)	E:16.9 ± 1.0\u00B0C:16.6 ± 0.9	30s (100%VO_2_peak)	30s (50%VO_2_peak)	8	12	①
Bogataj (29)	46 (22/24)	E:15.5 ± 0. C:15.7 ± 0.6	30s(NR)	15 s (Rest)	20	8	②
Cvetkovic (30) 2018	25 (11/14)	11–13	3 min (100%MAS)	3 min (Rest)	15	12	②
Farah (31)	19 (9/10)	E:15.4 ± 0.4\u00B0C:14.8 ± 0.4	30s (120%MAS)	30s (Rest)	NR	24	①
Koubaa (22)	29 (14/15)	E:13.0 ± 0.8\u00B0C:12.9 ± 0.5	2 min (80–90% MAS)	1 min (Rest)	NR	12	①
Boer (32)	32 (17/15)	E:18.0 ± 3.2\u00B0C:16.7 ± 3.6	15 s (100% VT)	45 s (50% VT)	10	15	①
Cao (33)	40 (20/20)	E:11.2 ± 0.7\u00B0C:10.9 ± 0.4	15 s (100%MAS)	15 s (50%MAS)	2	12	①
Cao (34)	25 (15/15)	E:11.4 ± 0.8\u00B0C:11.0 ± 0.7	15 s (90-100%MAS)	15 s (50%MAS)	2	12	①
Li (15)	32 (16/16)	11.0 ± 0.8	15 s (100-120%MAS)	15 s (50%MAS)	3	12	①
Cao (35)	40 (20/20)	11.0 ± 0.8	15 s (100-120%MAS)	15 s (50%MAS)	3	12	①
Yuan (36)	40 (20/20)	E:16.1 ± 1.2\u00B0C:15.9 ± 1.2	30s (100-110%MAP)	30s (50%MAP)	2–5	12	①

E, Experimental group; C, Control group; NR, Not reported; HRmax, Maximum heart rate; MAS, Maximum aerobic speed; VO2peak, Peak oxygen uptake; MAP, Maximum aerobic power; VT, Ventilatory threshold; ①VO2max; ②YO-YO test.

### 2.5 Quality assessment

The quality assessment of the included studies was conducted by two researchers independently, referring to the quality evaluation method revised based on the PRISMA principles by Buchheit (37). The included studies were categorized into three levels of risk: high risk (0–3 points), medium risk (4–6 points), and low risk (7–8 points) based on the cumulative evaluation score. The assessment included eight criteria: (1) clear inclusion criteria, (2) random allocation, (3) no significant baseline differences between groups, (4) blinding of outcome assessors, (5) all participants received the intended intervention or intention-to-treat analysis was performed, (6) dropout or lost to follow-up rate < 20% with detailed reasons, (7) sample size meeting the calculation requirements, and (8) reporting of effect size, precision, and results for each group (as shown in Table 2).

**Table 2:** Risk of bias evaluation of the included literature.

Literature	①	②	③	④	⑤	⑥	⑦	⑧	Total
Dias 2018	√	√	√	√	√	×	√	√	7
Ingul 2018	√	√	√	√	√	×	√	√	7
Kargarfard 2016	√	√	√	×	?	√	√	?	5
Murphy 2015	√	√	√	√	√	?	×	×	5
Starkoff 2014	√	√	√	√	√	√	√	×	7
Lau 2015	√	√	√	?	√	√	?	√	6
Racil 2013	√	√	√	?	√	√	×	√	6
Racil 2015	√	√	√	?	√	√	√	?	6
Bogataj 2021	√	√	√	√	√	√	?	√	7
Cvetkovic 2018	√	√	√	?	√	√	×	√	6
Farah 2014	√	√	√	?	√	×	×	√	5
Koubaa 2013	√	√	√	?	√	?	√	?	5
Boer 2013	√	√	√	?	√	×	√	×	5
Cao 2022(b)	√	√	√	√	√	×	√	√	7
Cao 2022(a)	√	√	√	√	√	√	√	√	8
Li 2023	√	√	√	?	√	×	√	√	6
Cao 2022	√	√	√	?	√	√	√	?	6
Yuan 2021	√	√	√	?	√	√	√	×	6
Total	18	18	18	7	17	10	12	10	

① Clear inclusion criteria were specified. ② Random allocation was performed. ③ There were no significant differences in baseline values between groups. ④ Blinding of outcome assessors was implemented. ⑤ All participants received the intended intervention or intention-to-treat analysis was performed on the experimental results. ⑥ The proportion of dropouts or lost to follow-up was less than 20%, and detailed reasons were provided. ⑦ The sample size met the calculation requirements. ⑧ The study reported the effect size, precision, and results for each group.

### 2.6 Data analysis

Review Manager 5.4 and Stata 17 software were used for the meta-analysis in this study. Since the included studies had continuous variables and inconsistent outcome measures, the statistical analysis was conducted using the standardized mean difference (SMD) and its 95% confidence interval (CI). A value of *p* < 0.05 was considered statistically significant. Additionally, Cochran’s Q test and the I^2^ statistic were employed to determine the magnitude of heterogeneity among studies: I^2^ < 25% indicates low heterogeneity, 25% < I^2^ < 50% indicates moderate heterogeneity, and I^2^ > 50% indicates high heterogeneity. A significance level of *p* < 0.05 indicates significant heterogeneity. Only when I^2^ < 50% and P > 0.05, a fixed-effects model can be used for analysis; otherwise, a random-effects model is applied. Egger’s test and funnel plots were used to assess publication bias among the included studies. If publication bias was detected, the trim and fill method was employed to assess the stability of the combined results. Additionally, sensitivity analyses were conducted using two methods to ensure the reliability of the results: (1) switching between random-effects and fixed-effects models and reanalyzing all statistical results, and (2) assessing the significant influence of each individual study by sequentially excluding one study at a time.

## 3 Results

### 3.1 Quality assessment results

After assessing the quality of the 18 included studies, it was found that 6 studies were classified as low risk (7–8 points), 12 studies were classified as medium risk (4–6 points), and no studies were classified as low quality (0–3 points). All 18 studies met the low-risk requirements in terms of inclusion criteria, random allocation, and baseline comparability. Regarding blinding, only 7 studies implemented blinding of outcome assessors, reducing the potential bias introduced by researchers’ subjective intentions. In terms of intervention implementation, only 1 study did not provide a detailed description of the intervention. Regarding dropout and description, all 18 studies reported the number of dropouts and the reasons, but 8 studies had dropout rates exceeding 20% of the original sample size. In terms of reporting results, 8 studies only reported the numerical values of outcome measures before and after the intervention, without reporting the effect size. Overall, the average risk of bias evaluation score for the included studies was 6.1, indicating a medium risk level. This suggests that future experimental studies should control dropout rates and conduct further statistical analysis on the changes in outcome measures, in addition to implementing blinding, to improve the methodological quality of study designs.

### 3.2 Effect of high-intensity interval training on cardiorespiratory fitness in children and adolescents with overweight or obesity

**Figure 2:**
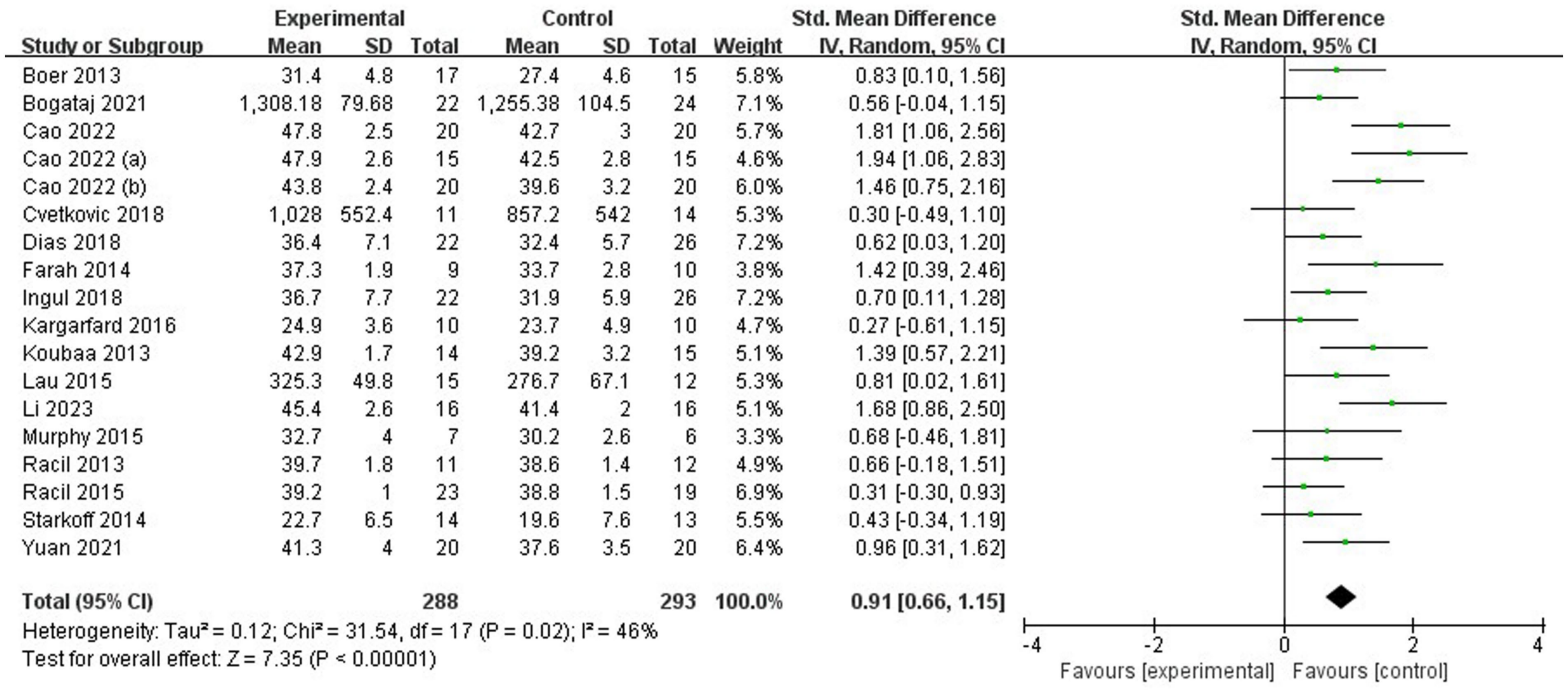
Meta-analysis forest plot.

First, a test for heterogeneity was conducted on the 18 included studies, revealing moderate heterogeneity (I^2^ = 46%, *p* = 0.02). Therefore, a random-effects model was used to examine the overall effect size. According to Cohen’s criteria, effect sizes of 0.2 indicate a small effect, 0.2–0.8 indicate a moderate effect, and above 0.8 indicate a large effect. The overall pooled effect size of the 18 studies was *d* = 0.91. The two-tailed test result (*p* < 0.00001) and the 95% CI (0.66, 1.15) both indicated statistical significance. These findings demonstrate that high-intensity interval training significantly improves the cardiorespiratory fitness level of children and adolescents with overweight or obesity (Figure 2).

### 3.3 Sources of heterogeneity

Based on the overall effect size analysis, it was observed that the included studies exhibited moderate heterogeneity (I^2^ = 46%, *p* = 0.02). To explore the sources of heterogeneity, this study conducted meta-regression analyses using intervention duration, number of intervention sets, load time, rest time, and load/rest time ratio as independent variables (as shown in Table 3).

**Table 3:** Overview of meta-regression results.

Explanatory variable	Regression coefficient	SE	Test statistic (*t*)	*P* > |*t*|	95%CI
Intervention period	0.13	0.14	0.89	0.389	−0.18,0.43
Intervention repetitions	−0.36	0.12	−3.01	0.011	−0.60,0.10
Load time	−0.01	0.50	−0.01	0.992	−1.10,1.09
Rest time	−0.31	0.53	−0.60	0.561	−1.46,0.83
Load/Rest ratio	0.03	0,18	0.15	0.881	−0.37,0.42
Intercept	1.67	0.64	2.58	0.024	0.26,3.06

The results of the meta-regression analysis indicate that the intervention duration, load time, rest time, and load/rest time ratio subgroups all have *p* values >0.05, indicating no significant differences. However, the subgroup analysis of the number of intervention sets showed a *p* value of 0.011 < 0.05, suggesting that the number of intervention sets is one of the sources of heterogeneity in this study. Additionally, based on the results of the meta-regression, a more detailed subgroup analysis can be conducted to explore the dose–response relationship of high-intensity interval training on improving cardiorespiratory fitness in children and adolescents with overweight or obesity.

### 3.4 Assessment of publication bias

**Figure 3:**
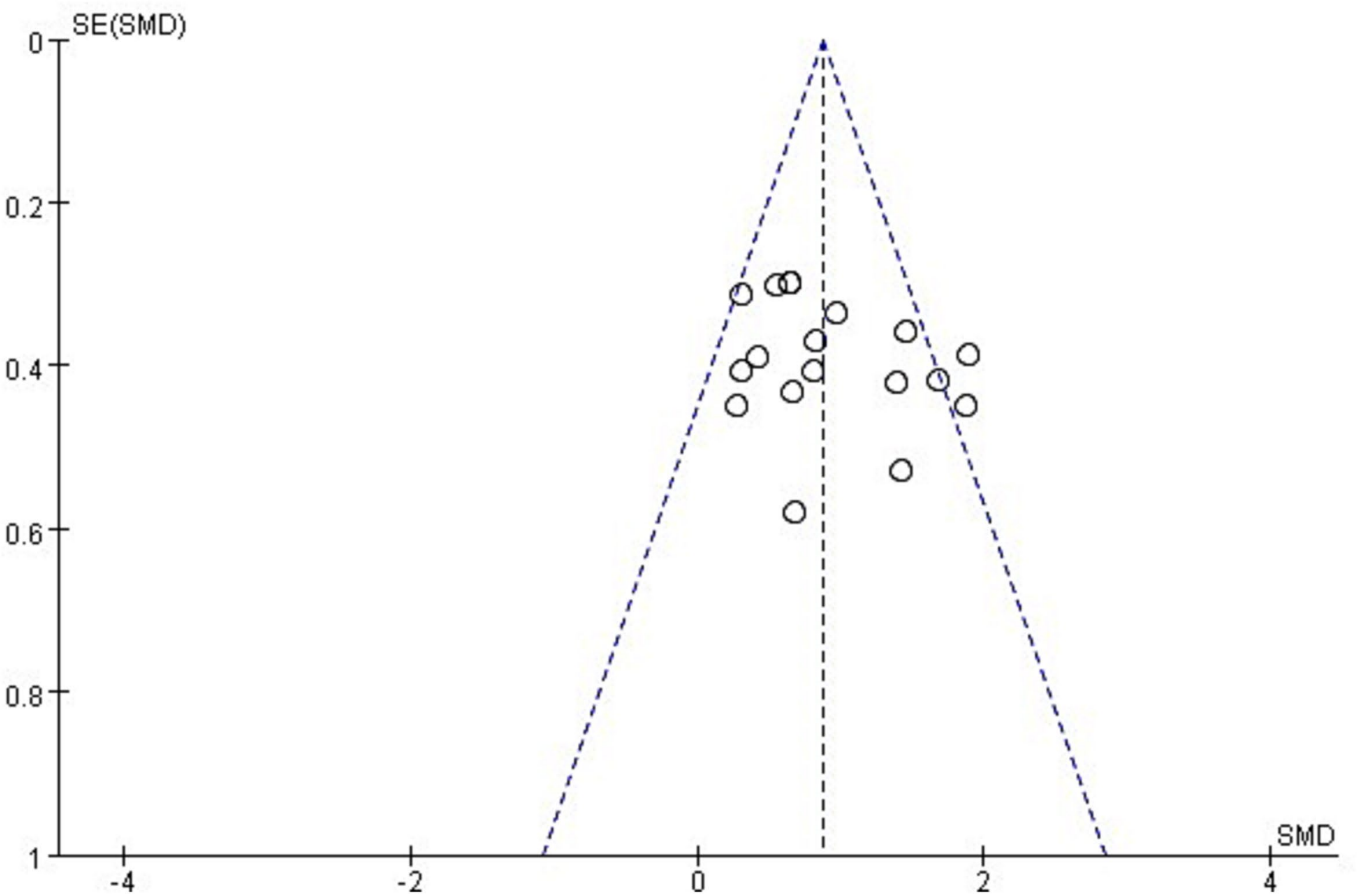
Funnel plot of publication bias.

**Figure 4:**
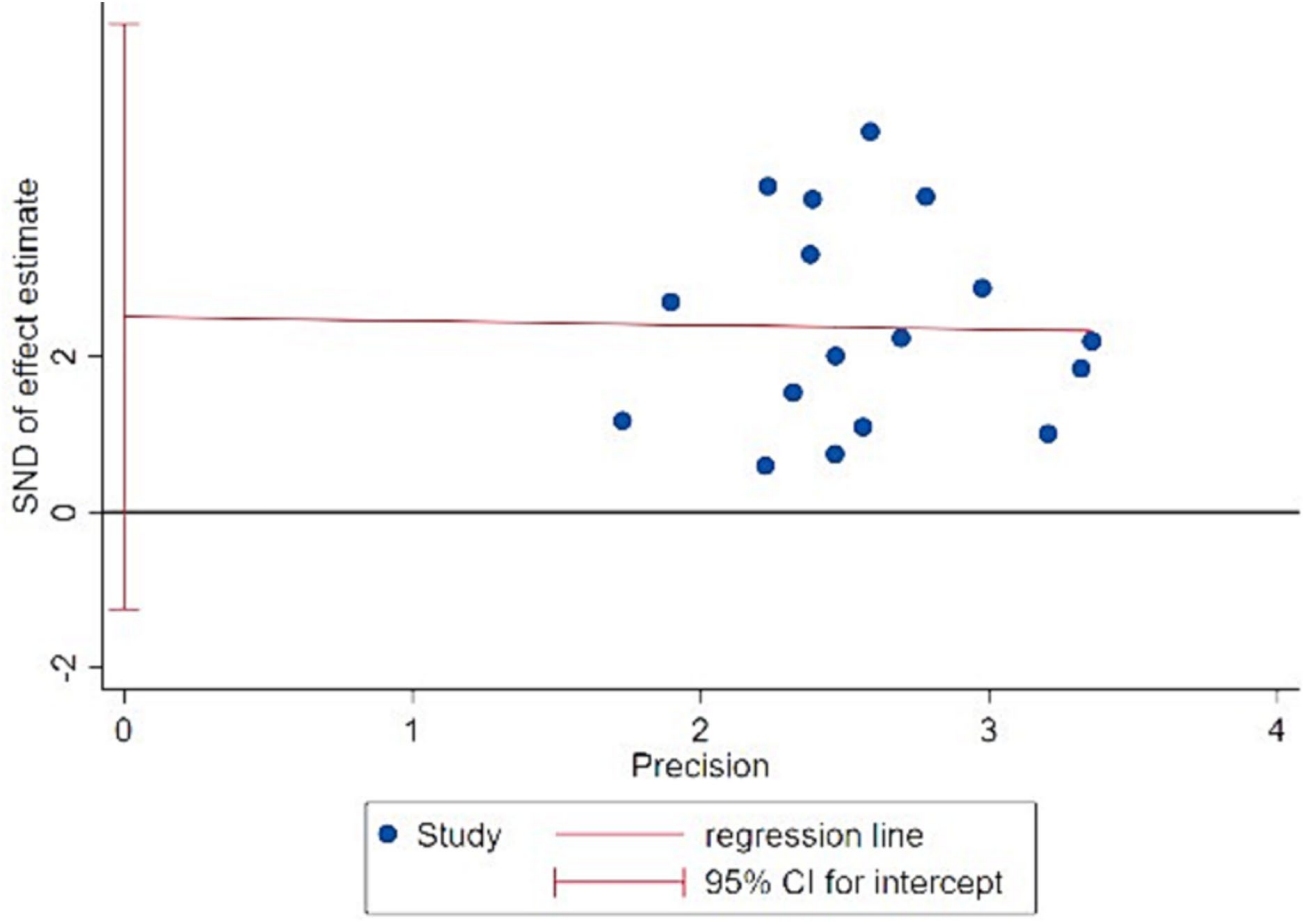
Egger test results chart.

To ensure the scientific rigor and validity of the research findings, an assessment of publication bias was conducted for the included studies. When the number of studies included in the meta-analysis exceeds 10, publication bias can be assessed using a funnel plot, along with Begg’s test and Egger’s test for quantitative evaluation. Firstly, based on the distribution of the 18 included studies in the funnel plot (Figures 3, 4), the results show that the effect sizes of most studies are evenly distributed on both sides of the average effect value, indicating a balanced distribution. Although there may be some slight bias toward the right side in a few individual studies, it is unlikely to have a significant impact on the overall results. Secondly, both Begg’s test (*Z* = 0.72, *p* = 0.472) and Egger’s test (*t* = 1.47, *p* = 0.161) yielded *p* values greater than 0.05. Therefore, it can be concluded that there is no apparent publication bias among the included studies, and the data analysis results are considered scientifically sound and rigorous.

### 3.5 Sensitivity analysis quality assessment results

To assess the reliability of this study, a sensitivity analysis was conducted by performing statistical analyses using both random-effects and fixed-effects models. The results showed that there were no significant changes in the statistical results after swapping between the two models. This indicates that the findings of the study are robust and not heavily influenced by the choice of the model.

Furthermore, a leave-one-out sensitivity analysis was conducted to examine the potential impact of each individual study on the overall results. The results showed that after removing each individual study, the range of the pooled effect size (SMD) was between 0.95 and 0.84, which still falls within the range of a large effect. The range of I^2^ was between 50 and 34%, and the *p* values were all less than 0.00001. This indicates that the removal of any single study had minimal impact on the overall effect size, and the results remained statistically significant. The analysis confirms the stability and reliability of this study.

### 3.6 Dose–response relationship of high-intensity interval training to improve cardiorespiratory fitness in children and adolescents with overweight or obesity

After confirming the significant positive effect of high-intensity interval training on cardiorespiratory fitness in children and adolescents with overweight or obesity, this study conducted a subgroup analysis to explore the dose–response relationship between high-intensity interval training and the improvement in cardiorespiratory fitness in this population. The analysis was conducted based on intervention duration, intervention frequency, single-bout exercise duration, single-bout rest duration, and load/rest ratio (Table 4). Since all included studies had an intervention frequency of 3 times per week, intervention frequency was not analyzed as a separate subgroup.

1. Intervention duration: in this subgroup, a total of 581 participants were included. The effect sizes among the three subgroups showed moderate heterogeneity (I^2^ = 57.9%), indicating that intervention duration has some influence on the relationship between high-intensity interval training and the improvement in cardiorespiratory fitness among children and adolescents with overweight or obesity. Specifically, high-intensity interval training with a duration of more than 10 weeks produced the largest effect size: SMD = 1.04, 95% CI (0.74, 1.34), *p* < 0.00001. The subgroup with an intervention duration of 4–8 weeks had the next highest effect size: SMD = 0.62, 95% CI (0.13, 1.12), *p* = 0.01. The subgroup with an intervention duration of 8–10 weeks had the smallest effect size: SMD = 0.46, 95% CI (−0.03, 0.95), *p* = 0.07, although it was not statistically significant.
2. Intervention frequency: in this subgroup, a total of 581 participants were included. The effect sizes among the three subgroups showed moderate heterogeneity (I^2^ = 49.2%), indicating that intervention frequency has some influence on the relationship between high-intensity interval training and the improvement in cardiorespiratory fitness among children and adolescents with overweight or obesity. High-intensity interval training with 2–8 intervention sets per session produced the largest effect size: SMD = 1.13, 95% CI (0.71, 1.15), *p* < 0.00001. The subgroup with 8–12 intervention sets had the next highest effect size: SMD = 0.80, 95% CI (0.47, 1.14), p < 0.00001. The intervention effect gradually decreased as the number of intervention sets increased, with the smallest effect size observed in the subgroup with more than 12 intervention sets: SMD = 0.57, 95% CI (0.23, 0.93), *p* < 0.001.
3. Single bout exercise duration: in this subgroup, a total of 581 participants were included. The effect sizes among the three subgroups showed high heterogeneity (I^2^ = 62.7%), indicating that single-bout exercise duration has some influence on the relationship between high-intensity interval training and the improvement in cardiorespiratory fitness among children and adolescents with overweight or obesity. The subgroup with a single bout exercise duration of 15 s to 1 min produced the largest effect size: SMD = 1.09, 95% CI (0.76, 1.42), *p* < 0.00001. The subgroup with a single bout exercise duration of 1–2 min had the next highest effect size: SMD = 0.84, 95% CI (0.22, 1.45), *p* = 0.008. The subgroup with a single bout exercise duration of 3 min or more had the smallest effect size: SMD = 0.53, 95% CI (0.19, 0.87), *p* = 0.002. The trend observed in the effect sizes of these three subgroups suggests that the intervention effect gradually decreases with longer single-bout exercise duration.
4. Single bout rest duration: in this subgroup, a total of 581 participants were included. The effect sizes among the three subgroups showed high heterogeneity (I^2^ = 51.3%), indicating that single-bout rest duration has some influence on the relationship between high-intensity interval training and the improvement in cardiorespiratory fitness among children and adolescents with overweight or obesity The subgroup with a single bout rest duration of 15 s to 1 min produced the largest effect size: SMD = 10.9, 95% CI (0.76, 1.42), *p* < 0.00001. The subgroup with a single bout rest duration of 1–2 min had the next highest effect size: SMD = 0.69, 95% CI (0.16, 1.21), *p* = 0.01. The subgroup with a single bout rest duration of 3–4 min had the smallest effect size: SMD = 0.54, 95% CI (0.08, 1.00), *p* = 0.02. The trend observed in the effect sizes of these three subgroups suggests that longer single-bout rest duration leads to weaker intervention effects.
5. Load/rest ratio: in this subgroup, a total of 581 participants were included. The effect sizes among the three subgroups showed high heterogeneity (I^2^ = 41.6%), indicating that the load/rest ratio has some influence on the relationship between high-intensity interval training and the improvement in cardiorespiratory fitness among children and adolescents with overweight or obesity. The subgroup with a load/rest ratio of 1 produced the largest effect size: SMD = 1.11, 95% CI (0.73, 1.50), *p* < 0.00001. The subgroup with a load/rest ratio less than 1 had the next highest effect size: SMD = 0.78, 95% CI (0.17, 1.40), *p* = 0.01. The subgroup with a load/rest ratio greater than 1 had the smallest effect size: SMD = 0.65, 95% CI (0.34, 0.96), *p* < 0.0001.

**Table 4:** Dose-effect relationship between high-intensity interval exercise pairs and cardiorespiratory fitness in children and adolescents with overweight or obesity.

Subgroup	Heterogeneity test			Category	sample size	effect size and 95%CI	Two-tailed test	
	*x* ^2^	*I* ^2^	*p*				Z	*p*
Intervention period				4 ~ 8 wk	67	0.62(0.13,1.12)	2.47	0.01
	4.75	57.9	0.09	8 ~ 10 wk	66	0.46(−0.03,0.95)	1.83	0.07
				over 10 wk	448	1.04(0.74,1.34)	6.76	<0.00001
Intervention repetitions				2 ~ 8 sets	320	1.13(0.71,1.55)	5.3	<0.00001
	3.94	49.2	0.14	8 ~ 12 sets	151	0.80(0.47,1.14)	4.67	<0.00001
				over 12 sets	133	0.57(0.23,0.93)	3.22	<0.001
Load time				15 s ~ 1 min	371	1.09(0.76,1.42)	6.39	<0.00001
	5.36	62.7	0.07	1 ~ 2 min	69	0.84(0.22,1.45)	2.66	0.008
				over 3 min	141	0.53(0.19,0.87)	3.06	0.002
Rest time				15 s ~ 1 min	371	1.09(0.76,1.42)	6.39	<0.00001
	4.11	51.3	0.13	1 ~ 2 min	89	0.69(0.16,1.21)	2.57	0.01
				3 ~ 4 min	100	0.54(0.08,1.00)	2.31	0.02
Load/rest ratio				ratio<1	45	0.78(0.17,1.40)	2.51	0.01
	3.43	41.6	0.18	ratio = 1	318	1.11(0.73,1.50)	5.65	<0.00001

## 4 Discussion

### 4.1 Analysis of the effect of high-intensity interval training on cardiorespiratory fitness in children and adolescents with overweight or obesity

Overall, high-intensity interval training has a significant positive effect on cardiorespiratory fitness levels in children and adolescents with overweight or obesity. This finding is consistent with previous meta-analyses and systematic reviews (38–40). Based on the synthesis of existing meta-analyses and relevant research evidence, this study concludes that high-intensity interval training has a positive effect on improving cardiorespiratory fitness levels in children and adolescents with overweight or obesity.

**Figure 5:**
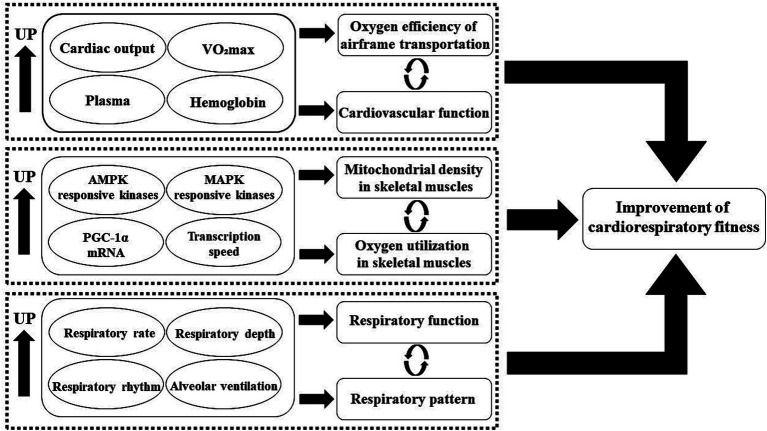
Mechanism of high-intensity interval training improving cardiorespiratory fitness in children and adolescents with overweight or obesity.

Regarding the mechanisms underlying the rapid improvement of cardiorespiratory fitness through high-intensity interval training, studies in the cardiovascular and skeletal muscle domains provide some insights (Figure 5). After high-intensity exercise, plasma parameters, maximal cardiac output, stroke volume, and hemoglobin levels all show significant increases. Research by Jan Helgerud (41) and Ulrik Wisløff (42) found that after a long period of HIIT training, subjects experienced an increase in cardiac output, and their maximal oxygen uptake (VO2max) was approximately 10% higher than the baseline. Furthermore, they observed significant improvements in plasma and hemoglobin levels as well as improved endothelial function in the HIIT intervention group, leading to enhanced oxygen transport efficiency and more efficient training effects in a shorter training time. Increased mitochondrial density and improved oxygen utilization in skeletal muscles may be another important reason for the significant enhancement of cardiorespiratory fitness through HIIT. In fact, related studies have confirmed that HIIT is more effective than traditional training methods in improving skeletal muscle oxidative capacity (43). From the perspective of the molecular adaptive mechanisms of skeletal muscle oxidative capacity, HIIT can activate the activity of AMPK and MAPK exercise-responsive kinases (44, 45), and increase the mRNA quantity of PGC-1α, a transcriptional factor that regulates mitochondrial oxidative function. This joint activation leads to increased transcription of mitochondrial genes and protein accumulation, resulting in the generation of more mitochondrial substances and enhancing the aerobic and anaerobic capacities of the body, ultimately improving cardiorespiratory fitness (46). In addition to the adaptive changes in the cardiovascular system and skeletal muscles, changes in ventilatory function also play a significant role in the improvement of cardiorespiratory fitness through HIIT. In intense exercise, increasing respiratory rate and depth are effective methods to obtain more energy supply. Compared to traditional training methods, HIIT can more closely simulate the respiratory rhythm and pattern of the body under high-intensity exercise scenarios, promoting adaptive improvements in ventilatory function and enhancing cardiorespiratory fitness. Studies by Ana Carolina Corte de Araujo in Brazil and Zu XiuMing in China have supported these observations. They found that obese children and adolescents who underwent HIIT interventions for more than 12 weeks showed significantly lower systolic and diastolic blood pressure and significant improvements in respiratory parameters, such as respiratory rate and alveolar ventilation (47, 48).

### 4.2 Analysis of the dose–response relationship of high-intensity interval training to improve cardiorespiratory fitness in children and adolescents with overweight or obesity

Based on the effects of intervention duration on the improvement of cardiorespiratory fitness in children and adolescents with overweight or obesity, interventions lasting more than 10 weeks had the largest effect size, followed by interventions lasting 4–8 weeks, and interventions lasting 8–10 weeks had the smallest effect size. Previous studies have shown that after a HIIT training period of more than 3 weeks, subjects’ cardiorespiratory function can improve by about 3 to 33% (49). As research on the optimal dose–response relationship of HIIT progresses, it has been suggested that the training period should be extended appropriately while ensuring that the total duration of each session is not less than 16 min, and the training intensity should be maintained above 7 METs (50). From the perspective of functional adaptation, long-duration high-intensity interval training can better adjust the high-energy phosphate bonds and reserves in skeletal muscles, leading to an improved oxidative potential. Considering that children and adolescents are still in the growth and development stage, their positive metabolic adaptations to physical exercise occur rapidly but have a relatively short duration. Therefore, this study suggests that high-intensity interval training lasting more than 10 weeks is likely to have the best effect on improving cardiorespiratory fitness in children and adolescents with overweight or obesity, given that other conditions are appropriate.

In terms of the number of intervention sets, 2–8 sets resulted in the largest effect size, while 8–12 sets and more than 12 sets had moderate effect sizes, showing a gradual decrease in effect size with an increasing number of intervention sets. It indicates that the intervention effect does not improve with an increasing number of sets. This may be because excessive exercise and alternating intervals can lead to fatigue in the sympathetic and parasympathetic nervous systems, weakening the central adaptation effect and reducing the intervention effect (14). Additionally, the baseline level of physical activity in most children and adolescents with overweight or obesity is not high, so increasing the number of intervention sets will significantly raise the exercise intensity. This not only fails to enhance their physical fitness and cardiorespiratory fitness but also may harm their health. Therefore, the results suggest that high-intensity interval training with 2–8 sets achieves the best effect on improving cardiorespiratory fitness in children and adolescents with overweight or obesity, and the number of exercise sets should be controlled to avoid excessive exercise.

Regarding the single-bout exercise duration and rest duration, the largest effect sizes were observed within the range of 15 s to 1 min, while durations of 1–2 min and 3 min or more resulted in moderate effect sizes, with a gradual decrease in effect size with longer durations. This indicates that longer exercise and rest durations do not necessarily lead to better results. Prolonged exercise and rest durations deviate from the essence of high-intensity interval training and make it more similar to traditional aerobic training, which hinders the rapid and effective recovery of the body and nervous system, consequently affecting the intervention effect. This is supported by the subgroup analysis of the exercise/rest duration ratio (14). When the ratio was 1:1, the intervention effect reached its maximum. It is evident that solely emphasizing exercise duration, rest duration, or intervention frequency is not scientifically appropriate for improving cardiorespiratory fitness in children and adolescents with overweight or obesity. Only when multiple influencing factors are synergistically adapted can an ideal intervention effect be achieved (38).

Although the meta-analysis suggests that high-intensity interval training interventions lasting over 10 weeks, with a frequency of 3 times per week, 2–8 sets per session, and a ratio of exercise duration to rest duration of approximately 1:1, may be the most effective for improving cardiorespiratory fitness in children and adolescents with overweight or obesity, caution should be exercised in generalizing these dose–response recommendations for the following reasons: (1) The number of included studies in this analysis was limited, and the risk of literature omission exists due to the subjective preferences and limited experience of the researchers. Further validation of the effectiveness of these results is required. (2) The included studies were conducted in different countries and regions, and the effects may vary due to factors such as different races, social backgrounds, cultural differences, and lifestyle habits among participants. Therefore, the broad application of these dose–response recommendations in improving cardiorespiratory fitness in children and adolescents with overweight or obesity needs to be further investigated through future research and experiments, considering controlling other intervention factors, to explore the effects of different intervention doses on cardiorespiratory fitness.

### 4.3 Limitations and shortcomings of the study health, physics and mathematics references

This study primarily investigated the optimal dose–response relationship of high-intensity interval training for improving cardiorespiratory fitness in children and adolescents with overweight or obesity. Although the study strictly followed the PRISMA guidelines, there are still some limitations and shortcomings: (1) It is possible that not all relevant previous studies were comprehensively and carefully searched and included. (2) Despite the absence of significant result biases based on publication bias tests and sensitivity analyses, there may still be potential risks of bias considering the limited number of included studies, the shortage of intervention methods, and the low proportion of high-quality studies. (3) Some included studies used the Level 1 YO-YO test to measure outcome indicators. Although this measurement method can provide the subjects’ VO2max values, the testing process may introduce uncontrollable biases, resulting in some errors in the results of this study.

## 5 Conclusion and recommendations

After conducting a meta-analysis of published literature to assess the impact and dose–response relationship of high-intensity interval training on cardiorespiratory fitness in children and adolescents with overweight or obesity, the study results indicate the following: (1) High-intensity interval training can significantly enhance cardiorespiratory fitness levels in children and adolescents with overweight or obesity. (2) The research suggests that, to achieve a substantial improvement in cardiorespiratory fitness in a short period, children and adolescents with overweight or obesity should engage in high-intensity interval training for more than 10 weeks, 3 times per week, with 2 to 8 sets each time. The single load time-to-interval time ratio should be maintained at around 1:1.

Additionally, based on the research results, we propose the following three recommendations: (1) Schools should provide students with sufficient space and time for physical activities, especially for children and adolescents with overweight or obesity, who require more attention. For instance, in regular physical education classes, beyond traditional activities, it is essential to integrate short-duration, multi-set, and enjoyable high-intensity interval training, considering the individual characteristics and developmental stages of overweight and obese children and adolescents. This can subtly help them enhance physical fitness and promote health. (2) After school, parents should collaboratively establish detailed plans for extracurricular physical exercises with the school and students. Encouraging and supervising students to complete the designated amount of daily physical exercise, alongside their academic tasks, will sustain and expand the positive impact of school physical education on improving students’ physical fitness. (3) In the future, there should be higher-quality and larger-sample experimental studies conducted in schools. Under the premise of ensuring methodological quality, designing more detailed and feasible experimental plans will help identify intervention strategies and long-term mechanisms to enhance cardiorespiratory fitness and overall health in overweight and obese adolescents.

## Data availability statement

The original contributions presented in the study are included in the article/supplementary material, further inquiries can be directed to the corresponding author.

## Author contributions

YD: Conceptualization, Data curation, Formal analysis, Project administration, Resources, Software, Validation, Visualization, Writing – original draft. XW: Funding acquisition, Methodology, Project administration, Resources, Supervision, Validation, Writing – review & editing.
